# Therapeutic value of the metabolomic active neurotransmitter isorhynchophylline in the treatment of spontaneously hypertensive rats by regulating neurotransmitters

**DOI:** 10.1515/tnsci-2020-0185

**Published:** 2021-11-01

**Authors:** Homood Alharbi, Mohammad Ahmad, Zhenhua Tian, Ruixue Yu, Yun Lun Li

**Affiliations:** Department of Medical Surgical Nursing, College of Nursing, King Saud University, Riyadh, Saudi Arabia; Department of Pharmaceutical Sciences, Traditional Chinese Medicine, Shandong University, Jinan, China

**Keywords:** isorhynchophylline, hippocampus, targeted metabolomics, hypertension, neurotransmitter

## Abstract

Hypertension is one of the most reported cardiovascular and cerebrovascular diseases with significantly high morbidity and mortality rates. This condition threatens the very existence of human beings. Numerous studies conducted earlier revealed the good therapeutic effect of isorhynchophylline on hypertension since the former regulates the metabolic disorders in neurotransmitters. However, the mechanism behind this action is yet to be deciphered. The current study followed the targeted metabolomics method to investigate the changes in the neurotransmitter level in the hippocampus of spontaneously hypertensive rats (SHRs) after the rats were treated with isorhynchophylline. The authors predicted the metabolic pathways involved in extensively modified neurotransmitters. Further, the expressions of metabolism-key enzymes in mRNA and protein levels were also determined. When treated with isorhynchophylline, it induced notably varying metabolomic profiles of the hippocampus in SHRs. Isorhynchophylline perturbed a total of seven extensively modified neurotransmitters as well as the primarily related pathways such as tyrosine and glutamate metabolism. An increase in the key metabolic enzymes such as DDC, MAO, COMT, TH, and DβH was observed in the SHR group, whereas their levels decreased after treatment with isorhynchophylline. The expression of GAD67 established cross-current validity. So, isorhynchophylline has been proved to have potential therapeutic value to treat hypertension via tyrosine and glutamate metabolism in the hippocampus. Further, the current study also opened new ventures to further investigate the working mechanism of isorhynchophylline in hypertension.

## 1 Introduction

Essential hypertension (EH) remains one such predominant cardiovascular disease that mostly affects middle-aged and elderly people globally. Further, EH is also deliberated as an important risk factor for cardiovascular and cerebrovascular diseases. WHO published a report in the year 2004, which stated that 7.5 million people succumb to death globally due to EH and it is also placed in the top ten position in terms of annual loss incurred upon leading a disability-adjusted life [1]. Being a complicated process, the pathogenesis of hypertension is a challenging process to accomplish and is inclusive of dysregulation of renin–angiotensin systems, immune system, and neurogenic system [2,3,4]. So, great attention is being paid to the molecular mechanism and treatment protocol of EH, in recent years. Numerous studies established that the central nervous system is a good regulator of blood pressure. The studies conducted earlier argued that the altered neurotransmitters and cytokines in the brain could relieve hypertension [5,6]. The brain has loads of neurotransmitters that act as base materials for the neuromodulation process [7]. So, it is critical to observe and investigate the changes that occur in neurotransmitter levels *in vivo* to understand the pathogenesis of hypertension and so pave the way for new targets for novel antihypertensive drugs.

Isorhynchophylline is traditionally used in Chinese medicine and is extracted from *Uncaria* sp. By modulating the calcium ion channel, promoting autophagy, and through Aβ induced neurotoxicity, isorhynchophylline prevents the occurrence of amnesia and vascular dementia [8,9]. The compound exhibits antagonistic activity at the 5-HT2A receptor due to oxindole moiety configuration [8,10]. Further, when isorhynchophylline was applied in rats treated with Aβ25–35, it improved cognition by arresting beclin 1-mediated autophagy, induced by neuronal apoptosis, to aggravate the degradation of neuronal cell alpha-synuclein in primary cortical neurons, N2a, PC12 cells as well as SH-SY5Y [10,11]. The studies conducted in recent times [7,8,9,10] established that isorhynchophylline performs as an antioxidant and anti-inflammatory agent. Further, it also exerts neuroprotective effects and regulates neurotransmitters. Although neurotransmitter metabolism is regulated by isorhynchophylline and the variations in neurotransmitter levels have a close relationship with hypertension pathogenesis, little or no research has been conducted so far upon antihypertensive mechanisms in isorhynchophylline-triggered neurotransmitter metabolism disorders.

Researchers aim to characterize the isorhynchophylline mechanism in the current study on the treatment of hypertension. Isorhynchophylline was administered to spontaneously hypertensive rats (SHRs) after which the hippocampus was extracted from the samples. This study used the UPLC-Q-extractive/MS-full mass scan technique, a standard qualitative and quantitative analysis method that can be used for endogenous neurotransmitters. The levels of different neurotransmitters in rats prior to and after the treatment were analyzed, followed by the investigation of coherent metabolic pathways.

## 2 Materials and methods

### 2.1 Chemicals and reagents

A total of seven neurotransmitters were procured from Sigma Aldrich (Milan, Italy) and were used as standards. The procured neurotransmitters were R grade standard l-phenylalanine, standard l-glutamate, standard l-tyrosine, epinephrine hydrochloride, homovanillic acid, l-glutamine, and γ-aminobutyric acid. Dr. Ehrenstorfer GmbH (Germany) provided the standard dopamine hydrochloride. Further, isochineine and isorhynchophylline of R Grade were procured from Sigma Aldrich (Banglore, India). SYBR Premix Ex TaqTM II (Thermo-fisher), PrimeScriptTM RT reagent Kit with gDNA Eraser (Perfect Real Time), and Trizol Total RNA Extraction Kit were procured from Thermo-Fisher (USA). R grade reagents indicate 99.5% purity.

### 2.2 Animal handling

In this study, six paired male Wistar-Kyoto (WKY) rats and 12 male-specific pathogen-free SHRs were maintained in the SPF animal laboratory. Each cage had 6 rats; the weight of SHRs was 176.76 ± 12.18 g and aged 8 weeks. The temperature of the laboratory was maintained at 22 ± 1°C, whereas the relative humidity was 52 ± 5%. After one week of acclimatization, random allotment of SHRs was performed for the SHR group (*n* = 6) as well as for the isorhynchophylline (IRN) group (*n* = 6). In the latter group, water solution was administered to the rats that were given isorhynchophylline monomer at 0.251 mg kg^−1^. The control group (6 WKY rats) that was normotensive and the SHR group were administered uniform amounts of physiological saline for four consecutive weeks with 6 days, a week. At the time of intervention, isorhynchophylline dosage was adjusted as per the weight of the rats, checked once a week. Before and after administration, the blood pressure of each rat was checked noninvasively from the tail artery, through the tail–tail method for 1–4 weeks. The protocol followed here was in line with the Guide for the Care and Use of Laboratory Animals. Further, appropriate approval was obtained from the Ethics Committee of the Institution. All the animals were anesthetized after 12 h of final administration using 1% pentobarbital sodium at a dose of 40 mg kg^−1^; the hippocampus tissues were extracted and stored at −80°C for further analysis.


**Ethical approval:** The research related to animals’ use has been complied with all the relevant national regulations and was reviewed and approved by the animal protection and use committee of Shandong University of Traditional Chinese Medicine.

### 2.3 Metabolomics based on LC-MS

The extracted hippocampus sample, measuring 15 mg, was cut into pieces and was blended with 5 μL of ascorbic acid (0.1 mg mL^−1^), 500 μL of acetonitrile–isopropanol–water (3:3:2, v/v/v), and 5 μL of IS solution. A high-throughput grinding machine was used to homogenize the mixture; then the mixture was centrifuged at 12,000 rpm at 4°C for 15 min. The resultant precipitate was re-dissolved using 400 μL of water–acetonitrile (96:4, v/v). After centrifugation, the supernatant was collected for LC/MS analysis. For the preparation of the working solution and internal standard, diazepam-d5 and vitamin C were dissolved in 1 mg mL^−1^ methanol:water blend at a ratio of 1:1, v/v. This method was also followed to obtain the reference standard solutions for eight neurotransmitters (1 mg mL^−1^). A standard curve was prepared for which l-glutamine, l-tyrosine, l-phenylalanine, and dopamine hydrochloride were diluted with IS and vitamin C at varying concentrations in the range of 100–5,000 ng mL^−1^. Likewise, l-glutamate and γ-aminobutyric acid were also diluted at varying concentrations in the range of 50–20,000 ng mL^−1^. UPLC-QExactive-MS system was used to quantify the neurotransmitters. The parameters mentioned in the literature [14] were considered for checking the accuracy of analysis and were validated with quality control samples. Once the LC/MS analysis was completed, principal component analysis (PCA) and orthogonal partial least-squares discriminant analysis (OPLS-DA) were performed for the metabolism datasets of samples.

### 2.4 Verification of molecular biology

The hippocampus samples, collected from different groups, were tested for determining the expression levels of rate-limiting enzymes such as tyrosine hydroxylase (TH), catechol-*O*-methyltransferase (COMT), dopamine β-hydroxylase (DβH), dopa decarboxylase (DDC), glutamate decarboxylase (GAD67), and monoamine oxidase (MAO) using RT-PCR. These enzymes are generally involved in neurotransmitter anabolism. The rest of the hippocampus samples were subjected to homogenization in liquid nitrogen, whereas total RNA was extracted after the application of Trizol reagents (Bao Bioengineering, Dalian, China).

Once the concentration and purity of RNA were verified using a spectrophotometer, the first strand cDNAs were subjected to reverse transcription with the help of a reverse transcription kit (Promega, Madison, USA). Table 1 lists the oligos and primers utilized in this study (manufactured by Sangon Biotech, Shanghai, China). Based on the instructions from the manufacturer, ELISA kits were used to detect the protein contents of six related enzymes. A total of nine biomarkers were used for pathway analysis in MetaboAnalyst 3.0 software (https://www.metaboanalyst.ca) to assess the extensively modified metabolic pathways, triggered by the intervention of isorhynchophylline, in the hippocampus of SHRs.

**Table 1:** The primer sequences of the target gene and internal reference

Primer	Sequence
DDC	5′-CTAGGGTGACAACCACGAAGAA-3′
	5′-TCTGTCCTGAGTTCCGGTATCT-3′
COMT	5′-TGGTGACTTTGTCCTGTAGGC-3′
	5′-CGCTACCTTCCAGACACACTT-3′
MAO	5′-GAACATCCTTGGACTCGGGTT-3′
	5′-ACTTCTGTGGCTCTTCTCTGC-3′
GAD67	5′-ACATTGTCAGTTCCAAAGCCAAGCG-3′
	5′-GCAACCGCAGGCACGACTG-3′
DβH	5′-GGAAGCGGACGGCAGAGGT-3′
	5′-GTTGTCATCACCACGGAGGCAG-3′
TH	5′-CTTCACAGAGAATGGGCGCT-3′
	5′-GCATAGGGTACCACCCACAG-3′

### 2.5 Bioinformatics and statistical analysis

SPSS 17.0 software was used to conduct the statistical analyses for the current study. All the experiments were conducted in triplicates and the results are expressed as mean ± standard deviation. Analysis of Variance (ANOVA)-LSD was conducted to execute multiple comparisons if the data are normally distributed; otherwise, the Kruskal–Wallis test was applied after the Dunnett T3 test. If *P* < 0.05, then the outcome was said to be statistically different.

SIMCA-P software (v 13.00, Unietrics, Umea, Sweden) was used to process the statistically significant neurotransmitter-targeted metabolomics data, saved in .xls format for multivariate statistical analysis. Once the data were scaled-up, the orthogonal-partial least-squares (OPLS-DA) method was used to distinguish the two groups, while the parameters *R* ^2^ *Y* and *Q* ^2^ were used to assess the model’s validity. Simultaneously, variable projection importance (VIP > 1) was used as a screening criterion to determine the neurotransmitter metabolites that significantly differentiated the groups.

## 3 Results

### 3.1 Blood pressure level

**Figure 1:**
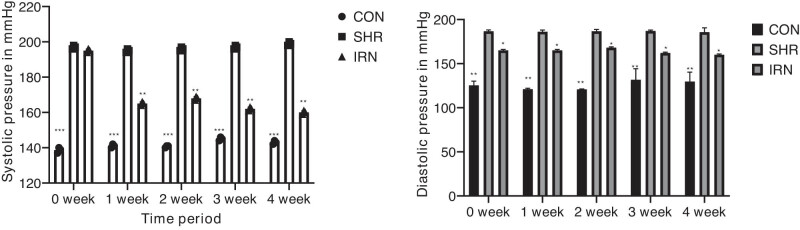
The systolic and diastolic blood pressure were recorded before and after 1–4 weeks of intervention (^*^ *P* < 0.05, ^**^ *P* < 0.01, ^***^ *P* < 0.001 compared to the SHR group).

The rats were checked for systolic and diastolic pressure to determine the impact of isorhynchophylline on the blood pressure of SHRs. Figure 1 shows that the SHR group rats exhibited high systolic and diastolic pressure compared to control group rats. When intervened with isorhynchophylline, a significant decline was observed in blood pressure (all *P* < 0.05) in SHR group rats. Thus, the study results indicate that isorhynchophylline inhibits hypertension to a significant level.

### 3.2 Targeted metabolomics analysis

**Figure 2:**
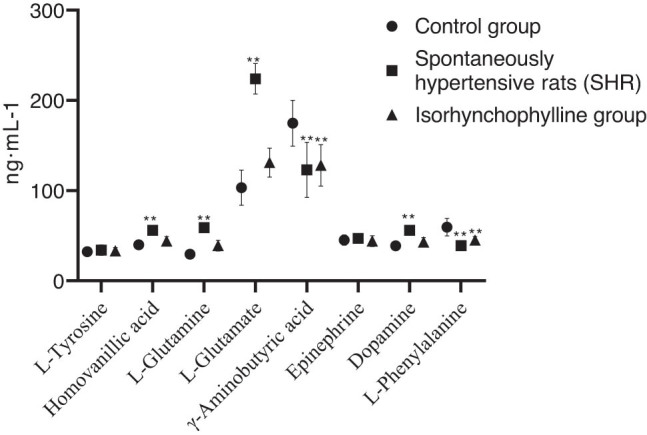
Quantitative and statistical results of neurotransmitters in the hippocampus.

**Figure 3:**
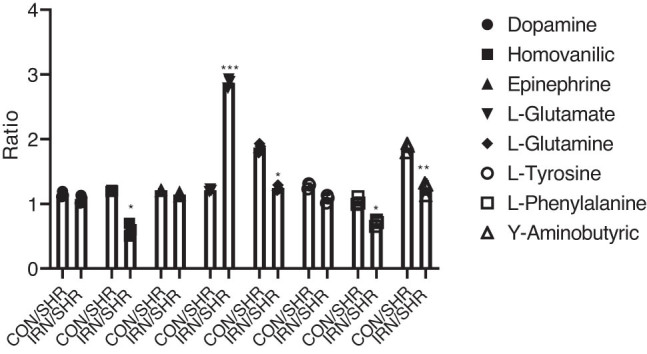
Potential biomarkers discovered by the VIP value in hippocampus (^**^ *P* < 0.01, ^**^ *P* < 0.05, ^***^ *P* < 0.001 compared to the SHR group).

**Figure 4:**
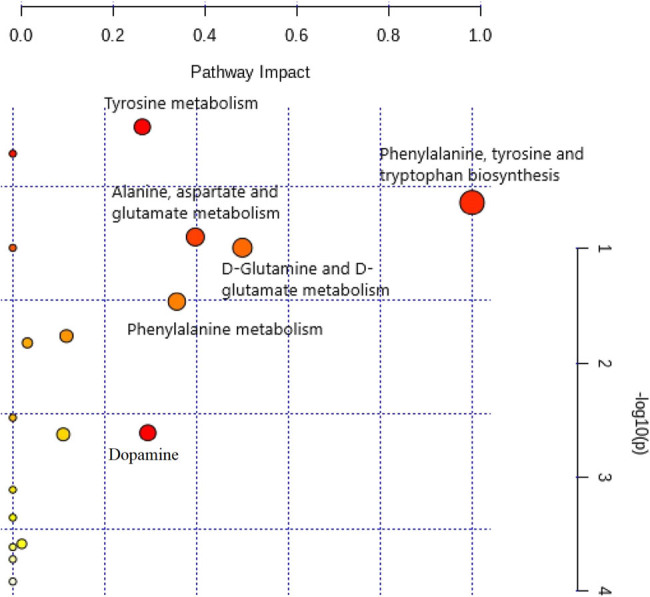
Pathway analysis with MetaboAnalyst software.

Figure 2 shows the quantitative (ng mL^−1^) and statistical results of eight neurotransmitters. In comparison with the control group, there was a significant increase observed in the expression levels of glutamine, dopamine, homovanillic acid, and glutamate in the SHR group. In parallel, the same group, i.e. SHR, exhibited a notable decline in γ-aminobutyric acid as well. There was a significant reversal observed in the expression levels of neurotransmitters in the SHR group after treating with isorhynchophylline; however, the same effect was not for γ-aminobutyric acid. They also showed a tendency to revert to normal levels. As per the indication from the unsupervised PCA model, every group had six specimens clustered together. This denotes that each group’s specimens possess similar characteristics. The OPLS-DA model was developed and portrayed to understand how groups were discriminated. From the clear cluster of WKY and SHR group samples, it can be understood that there existed a notably differential metabolism profile in the hippocampus tissues between SHRs and controls. Likewise, the clear demarcation between IRN and SHR groups infers that the metabolic changes observed in SHRs are the result induced by the isorhynchophylline treatment. For cross-validation, permutation tests were performed, whereas the model parameters such as *R* ^2^ *X* and *R* ^2^ *Y* were within the requirements. This outcome infers the presence of a reliable difference between the groups. As per variable importance in projection (VIP) values >1.0, 7 out of 8 substances in the hippocampus were regarded as potential metabolic biomarkers (Figures 3 and 4).

### 3.3 Molecular biology results

**Figure 5:**
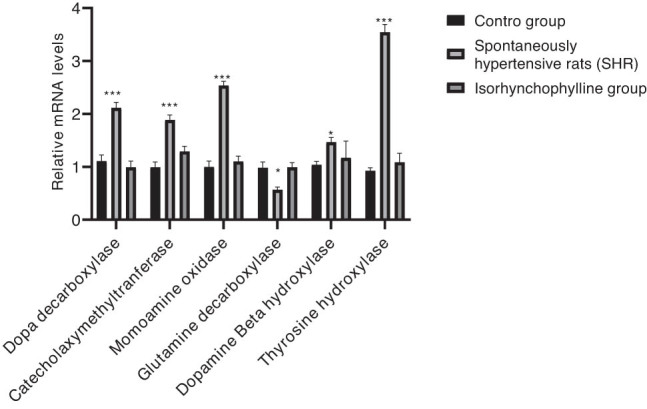
The relative mRNA levels of metabolism-key enzymes in the hippocampus (*n* = 6) were evaluated using the RT-PCR method. Data are expressed as mean ± SD. ^*^ *P* < 0.05 and ^**^ *P* < 0.01 compared to the control group.

**Figure 6:**
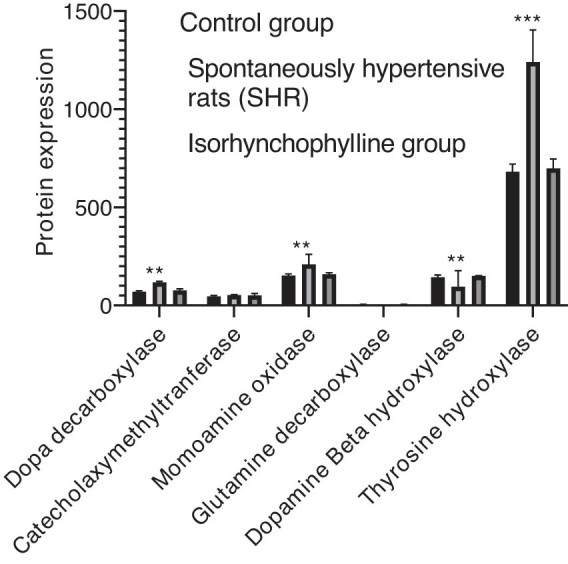
Validation of the diverse protein expressions in the hippocampus (*n* = 6). Data are expressed as mean ± SD (^*^ *P* < 0.05, ^**^ *P* < 0.01 compared to the control group).

From the results, it can be observed that there were high DDC, MAO, COMT, TH, and DβH levels in the control and IRN groups compared to the SHR group. However, a *vice versa* trend was observed in the case of GAD67 alone (Figure 4). Similar results were exhibited by the IRN group samples than the control group in terms of expression levels of all metabolism-key enzymes (Figure 5). Figure 6 shows similar protein expression patterns of metabolism-key enzymes.

## 4 Discussion

Various risk factors contribute to the occurrence of cardiovascular diseases among which hypertension is the most important one and has close ties with high mortality. Isorhynchophylline is an established compound known for its antihypertensive property, whereas its antihypertensive mechanism has a proven track record due to multichannel synergy. But, no studies have been conducted so far regarding its neuropathological mechanism. The current study followed the LC/MS-based metabolomics method to track the modifications in neurotransmitters when the hippocampus of SHRs was treated with isorhynchophylline. The results retrieved from quantitative analysis and OPLS-DA analysis of brain tissue samples from SHRs indicate that there exists a significant association between essential hypertension and the seven neurotransmitters including monoamine- and amino acid neurotransmitters. A disturbance was observed in both tyrosine and glutamate metabolism pathways. In this scenario, it is obvious that the expression profiles of six pathway-related key enzymes were altered in SHRs. However, when administered with isorhynchophylline, these alterations could be reversed. Thus, isorhynchophylline has been proved to protect SHRs from hypertension primarily through tyrosine and glutamate metabolism pathways.

In the current study, low content of tyrosine was found in the hippocampus of SHR group rats compared to WKY rats, although it was insignificant, and changes might be attributed to catecholamine disorder in the SHR group [12]. Being a rate-limiting enzyme, TH is held accountable for the biosynthesis of catecholamine. TH plays a key role in the first process of catecholamine biosynthesis, i.e., catalyzing the synthesis of levodopa, a biosynthetic precursor of catecholamine, through tyrosine [13]. When levodopa gets accumulated, it aggravates the synthesis of catecholamines in addition to increased activities of DDC, DβH, MAO, and COMT [14]. In general, catecholamines enact an important role in increasing blood pressure since it damages the structure of neuronal cells through sympathetic activity [15]. When treated with isorhynchophylline, a significant decline was observed in catecholamine levels in hippocampus tissues of SHRs. This confirms the anti-hypertension effect of isorhynchophylline via regulating the biosynthesis of catecholamines. Glutamate and γ-GABA play crucial roles in blood pressure regulation by the maintenance of brain functions [16]. Glutamate, being an excitatory neurotransmitter, tends to activate RAAS when it is in high quantity and increases blood pressure [17]. GAD67 is generally involved in transforming glutamate to γaminobutyric acid. Being a suppressive neurotransmitter, γ-GABA is present in nerves and is sedative in nature when antineurons are hyperactive [15,16,17].

## 5 Conclusion

As per the current study outcomes, in comparison with control rats, a significant increase was observed in glutamate and glutamine levels when induced by hypertension and a decline was noticed in the γ-GABA level in the hippocampus of SHRs. However, when administered isorhynchophylline, these changes were reversed. An earlier study by Li et al. indicated the association of the antihypertensive effect of isorhynchophylline with tyrosine and glutamate metabolism in the hypothalamus, whereas in the present study we see isorhynchophylline treatment reduced blood pressure in SHRs through tyrosine and glutamate metabolism pathways in the hippocampus. Therefore, we can conclude that there are similar pathways from both brain regions involved in the antihypertensive effect of isorhynchophylline [18]; however, further work is required to ensure which part of the brain dominates the tyrosine and glutamate metabolism in association with the antihypertensive effect of isorhynchophylline. To conclude, the study outcomes reveal that tyrosine and glutamate metabolism is the main pathways, hindered by the intervention of isorhynchophylline, in the hippocampus region of the SHR group.
